# Health effects of Indigenous language use and revitalization: a realist review

**DOI:** 10.1186/s12939-022-01782-6

**Published:** 2022-11-28

**Authors:** D. H. Whalen, Melissa E. Lewis, Stefanie Gillson, Brittany McBeath, Bri Alexander, Kate Nyhan

**Affiliations:** 1 Endangered Language Fund, 300 George St., Suite 900, New Haven, CT 06511 USA; 2 Department of Family & Community Medicine, University of Missouri School of Medicine, MA301 Medical Sciences Bldg, Columbia, MO 65212 USA; 3 Yale Child Study Center, 230 South Frontage Road, New Haven, CT 06520 USA; 4 School of Kinesiology and Health Studies, Queen’s University, 28 Division Street, Kingston, ON K7L 3N6 Canada; 5 Program in Anthropology, CUNY Graduate Center, 365 Fifth Avenue, New York, NY 10016 USA; 6 Cushing/Whitney Medical Library, 333 Cedar St, New Haven, CT 06510 USA

**Keywords:** Language use, Language revitalization, Health, Indigenous, Realist review

## Abstract

**Background:**

Indigenous populations across the world are more likely to suffer from poor health outcomes when compared to other racial and ethnic groups. Although these disparities have many sources, one protective factor that has become increasingly apparent is the continued use and/or revitalization of traditional Indigenous lifeways: Indigenous language in particular. This realist review is aimed at bringing together the literature that addresses effects of language use and revitalization on mental and physical health.

**Methods:**

Purposive bibliographic searches on Scopus were conducted to identify relevant publications, further augmented by forward citation chaining. Included publications (qualitative and quantitative) described health outcomes for groups of Indigenous people who either did or did not learn and/or use their ancestral language. The geographical area studied was restricted to the Americas, Australia or New Zealand. Publications that were not written in English, Spanish, French, Portuguese or German were excluded. A realist approach was followed to identify positive, neutral or negative effects of language use and/or acquisition on health, with both qualitative and quantitative measures considered.

**Results:**

The bibliographic search yielded a total of 3508 possible publications of which 130 publications were included in the realist analysis. The largest proportion of the outcomes addressed in the studies (62.1%) reported positive effects. Neutral outcomes accounted for 16.6% of the reported effects. Negative effects (21.4%) were often qualified by such issues as possible cultural use of tobacco, testing educational outcomes in a student’s second language, and correlation with socioeconomic status (SES), health access, or social determinants of health; it is of note that the positive correlations with language use just as frequently occurred with these issues as the negative correlations did.

**Conclusions:**

Language use and revitalization emerge as protective factors in the health of Indigenous populations. Benefits of language programs in tribal and other settings should be considered a cost-effective way of improving outcomes in multiple domains.

**Supplementary Information:**

The online version contains supplementary material available at 10.1186/s12939-022-01782-6.

## Background

Since the start of settler colonization, Indigenous^1^ languages have been declining in use and number of speakers. Acts of genocide, ethnocide, and assimilation play roles in this decline, including recent examples to limit Indigenous language use through policy (e.g., [1, 2]) or by adoption (forced or voluntary) of a regionally dominant language [3]. Many groups have reacted to this loss by engaging in a variety of language revitalization techniques, ranging from pairing younger learners with elder speakers (“master/apprentice programs” [4]) to recreation of languages without current speakers based on archival material [5]. Those latter efforts have led to a shift from calling languages “dead” to “sleeping” [6, 7]. If a language is facing decline or in need of revitalization, the task to revitalize is quite challenging [8, 9].

Despite the challenges facing revitalization, an ever-increasing number of Indigenous communities throughout the world are engaging in that work. The most commonly cited example of successful revitalization is that of Hebrew [10, 11], but other major efforts have been found for Welsh [12], Māori [13] and Hawaiian [14]. Such efforts are directed toward increasing the use of the language, but the efforts also serve one or more of several larger goals: sovereignty, cultural reclamation, community cohesion, identity, and cultural knowledge transmission (e.g., [15]). Indeed, although higher levels of proficiency and broader community use are often taken as the hallmarks of success, these will not be the goals of every revitalization program, and therefore other vitality models based on more realistic community goals are largely absent and urgently needed. In the current realist review, revitalization was understood as language use defined by the community without regard for proficiency level.

One somewhat unexpected benefit attributed to such programs is an improvement in health. As outlined in our previous adventitious survey [16], language maintenance or revitalization has been found to have health benefits for a broad range of issues, such as suicide, obesity, diabetes, and educational performance. The present realist review updates and expands that work. Several years have passed since that review, and as could be expected, additional relevant results have been published. While the 2016 paper excluded mental health studies, those will be included in this review in order to give a fuller picture of health outcomes.

The realist review methodology [17–19] is an appropriate format for this topic: The results are scattered across publications and address many health issues, and there are too few that address a single health issue to justify a systematic review. The realist review process is similar to systematic and scoping reviews but allows flexibility of the search guidelines to best obtain manuscripts for this search. It is an appropriate technique for studying emerging issues which are not well covered by individual search terms. This approach uses database searches coupled with citation chaining, allowing for discovery of studies that are related to the cited articles even if they do not share any discoverable search terms.

Our hypothesis was that language use or revitalization will improve health on a wide range of measures. The mechanism is unlikely to be evident in the sparse literature that exists, but plausible candidates are increased social connections, increased sense of belonging and purpose, return to traditional food, and increased physical activity related to traditional activities. Further, Indigenous cultures, and therefore languages, have inherent health and well-being promoting principles that have co-evolved with natural environments for thousands of years [20]. From principles of traditional ecological knowledge to traditional healing methodologies, Indigenous language is the vessel that most efficiently carries these cultural lifeways. Further, Indigenous languages carry values that are health-promoting, including traditional foods practices and consumption, activities (exercise) to participate in, community relationship, and spiritual practices; these all relate to positive health outcomes. While mechanisms of health promotion will not be the focus of this study, future studies should work to collaborate with Indigenous culture keepers to learn about these mechanisms.

Articles available in searchable databases will largely use western definitions of health, but Indigenous definitions of health can vary by tribal community [21–25]. Although definitions vary across tribes, many Indigenous communities consider several aspects of health such as physical, mental, emotional, and spiritual as commonly seen in the medicine wheel [26]. Other critical aspects of health include, but are not limited to, community and social connections, tribal and cultural connection, connection to land and traditional lifeways, as well as resilience in the face of stress, oppression and discrimination [25, 27, 28]. Western reports, on the other hand, are often focused on specific illness and diseases and negative conditions that would present for treatment in western, clinical settings. Even data that fit such definitions may have different cultural implications, such as tribal acceptance of teenage pregnancy as “as an expectable life event rather than as a social problem to be eradicated” ([29]: 77). Therefore, both western and Indigenous definitions of illness are included. However, future studies may well take greater account of cultural definitions of health and purposeful sampling. It is worth noting that four of the six authors of this review are themselves Indigenous and bring that perspective to our study to the extent possible.

The circumstances for language maintenance vary greatly, but a broad distinction between first-language speakers (L1) and second-language learners (L2) is expected. Although both quantitative and qualitative studies will be included, it is to be expected that qualitative reports based on self-report are likely to have a positive response bias [30, 31]. In addition, we found that many qualitative studies did not address a specific disease process, being more focused on overall well-being; the exception is our category of education, where there were 7 such studies. The results of this realist review should inform the design of more direct studies, including prospective ones.

## Methods

Maintenance and revitalization of Indigenous languages are not intrinsically framed as health interventions. Maintenance, in particular, allows for a continuation of linguistic practices (thereby avoiding language extinction), while revitalization must often find ways of reintroducing language, sometimes even from historical records. It is the use of a language despite the breakdown in typical language transmission and/or pressure to adopt a majority language. Revitalization is a relatively new process, as reintroduction of a traditional language based on a cohort of current speaker and/or on historical records has not been necessary or feasible until modern times. The studies surveyed here are therefore ones that report health outcomes for both kinds of Indigenous language situations: where the language is still being transmitted as a first language and/or where the language is being revived. Some languages may have aspects of both language techniques for different segments of the community, but this level of detail is missing from the published reports. Some of the studies explicitly examine language as an issue, but many have the issue of language use embedded within them. In the latter case, correlations between language and health may not be remarked upon in the report itself. Thus many of the studies do not report on a “complex service intervention” as defined in Pawson et al. [18], but the correlational analyses allow us to gauge the effects of language use indirectly.

Some of the studies in our preliminary publication [16] and additional searches were in grey literature, and many were poorly indexed by simple search terms. Health outcomes have been found for a broad range of diseases and conditions, and some relevant papers will appear in search results set only if a keyword for the specific disease process at hand (such as “suicide” or “diabetes”) is used. It was not possible to list and search for every health issue that may have been studied in relation to Indigenous language use. Similarly, there is no search strategy, hedge, or filter to comprehensively retrieve papers about worldwide Indigenous communities, and indeed no single database in which that literature is comprehensively collected and fully searchable [32]. Therefore, the search of publication databases by keywords alone was inadequate for finding relevant resources. This review thus relies more heavily on citation chaining than other reviews (cf. [18]: 29), but citation chaining (also described as “snowballing”) is recognized as a valuable and productive technique in realist reviews [33].

### Search methods and criteria for identification of studies

The search of the bibliographic databases was conducted by a medical research librarian in collaboration with the corresponding author. Seeding of the search came from references previously reported [16] or identified by study personnel during further research. Controlled vocabulary and keywords were used in two Scopus searches (Table 1), one before and one after citation chaining. We chose to use Scopus, without using smaller specialized bibliographic databases such as iPortal or Native Health Database, for several reasons. First, we are confident that our “searching plus citation chaining” approach performs better than a “searching-only” approach. Second, Scopus contains more content, is more frequently updated, and has robust data export options. Forward citation chaining was performed using citationchaser on the researcher-supplied references [34]. This software relies on the citation graph of the Lens database. So, while we searched only one bibliographic database, documents that are not indexed in Scopus could nevertheless be identified by our information retrieval process.

**Table 1:** Database searches

Scopus search, 2021-11-08	
((TITLE-ABS-KEY ((indigenous OR indigeneity OR “first nation*” OR “native american*”) W/5 (language* OR linguist* OR speaker*))) OR (TITLE ((indigenous OR indigeneity OR “first nation*” OR “native american*”) AND (language* OR linguist* OR speaker*)))) AND ((TITLE-ABS-KEY (health* OR well-being OR wellbeing OR disease* OR nursing)) OR (SUBJAREA (deci OR immu OR medi OR neur OR nurs OR phar OR psyc OR heal OR mult)))	
740 results	
Scopus expanded search, 2021-11-17	
((TITLE-ABS-KEY ((indigenous OR indigeneity OR “first nation*” OR “native american*” OR aboriginal* OR metis OR inuit OR maori) W/5 (language* OR linguist* OR speaker*))) OR (TITLE ((indigenous OR indigeneity OR “first nation*” OR “native american*” OR aboriginal* OR metis OR inuit OR maori) AND (language* OR linguist* OR speaker*)))) AND ((TITLE-ABS-KEY (health* OR well-being OR wellbeing OR disease* OR nursing OR educat* OR graduat*)) OR (SUBJAREA (deci OR immu OR medi OR neur OR nurs OR phar OR psyc OR heal OR mult)))	
2194 results	

Each article selected for the title/abstract screening round was examined by two out of the five reviewers, randomly assigned. Thus each reviewer examined approximately 1400 abstracts. Conflicts between these two reviewers were resolved following a consensus approach (MEL, SG, DHW). The following inclusion and exclusion criteria were used. Inclusions were 1) Quantitative or qualitative report of health outcomes (Health outcomes include physical and mental issues, and “wellness” broadly defined; graduation rates/school performance are also health outcomes); 2) Indigenous language use, either maintenance by first-language speakers or revitalization (learning by second-language speakers), was related to health outcome; 3) Population in the Americas, Australia or New Zealand. Exclusions at the title and abstract screening stage were grouped into following reasons: 1) no health outcome was reported, 2) language use could not be related to the health outcome, 3) article focused on geographic areas outside of the target, 4) article not published in English, Spanish, French, Portuguese or German, and 5) other reasons.

In order to be included, the publications must have described correlations between language use and health outcomes. Both quantitative and qualitative studies were accepted, with “qualitative” including “eyewitness” accounts [35] describing personal (often self-reflective) observations of either specific or global effects for individuals or groups. At the title and abstract stage, records were only excluded if the two screeners were confident that they did not meet the criteria. Unclear cases went on for more screening.

The articles initially selected for full-text screening were screened in detail for all inclusion and exclusion criteria. The full-text screening round was conducted by a primary reviewer (DHW) and one of the four other reviewers (MEL, SG, BM, BA). Conflicts were resolved following a consensus approach (DHW, MEL, SG). Final data extraction was initially performed by one reviewer (DHW) and validated by the other original reviewer of that article.

Non-peer-reviewed documents (e.g., conference papers, government reports, etc.) were included if they seemed to have original research. For example, masters theses and dissertations were included but periodicals such as newspapers and magazines were not. No authors were contacted.

Data categories were derived from an estimation of the most useful way to organize the rather disparate results. Some topics were typical labels for health issues, such as diabetes, suicide or obesity. Even there, some subcategories were found, such as suicidal ideation or weight control. Other broad categories, such general health or education, had multiple subcategories that still seemed more appropriate to consider together. Topics that appeared in a single study and did not seem to belong to one of the (emergent) broad categories were listed as “Other.” Both the broad, “overall,” category and the subcategories are listed in Table 2.

**Table 2:** Categories used for grouping results (“overall category”) and the more specific descriptors used in the studies (“subcategories)

Overall Category	Subcategories	Overall Category	Subcategories
general	protective factor	tobacco	smoking/cigarettes
	community well being		smokeless
	connectedness	suicide	suicide
	doctor visits		suicidal ideation
	happiness	mental	stress
	health		risky behaviors
	identity		resilience
	quality of life		anxiety
	well-being		self-esteem
	wellness	crime	violence victim
	physical activity		arrest
	cardiovascular	other	mammographic density
	poverty		English cognitive test
education	education		[not clear]
	graduation		various
	English reading		[null]
	Spanish testing		cancer
	academic achievement		sexual health
	school attendance		preterm birth
drug/alcohol	alcohol		syphilis
	illicits		oral health
diabetes			arthritis
obesity	obesity		condom use
	weight		parent report of language problems
			fat in diet
			nutrition

**Health outcomes** were classified broadly, with many studies using separate terms for potentially equivalent outcomes (“well-being,” “good health,” “general fitness,” “protective factor,” and others being examples). These are listed in full in Table 2.

**Quant/Qual** is a binary choice for studies. Note that some publications have multiple studies, and sometimes there are examples of both quantitative and qualitative studies in the same publication.

**Pos/Neut/Neg** is a three-way distinction for the effect of Indigenous language use on the health outcome: positive, neutral or negative. Some studies were coded as neutral when members of one group (e.g., males) had a negative outcome and another (in this example, females) had a positive one. Most of the statistical analysis within the selected manuscripts reported results for the entire population. However, for reasons of brevity, some minor results were not represented in our assessments, such as when multiple groups are assessed. In those cases, as now explained, a positive or negative result would be reported if the minor category was neutral. If both a positive and a negative result obtained, the overall categorization was “neutral.”

**Fig. 1:**
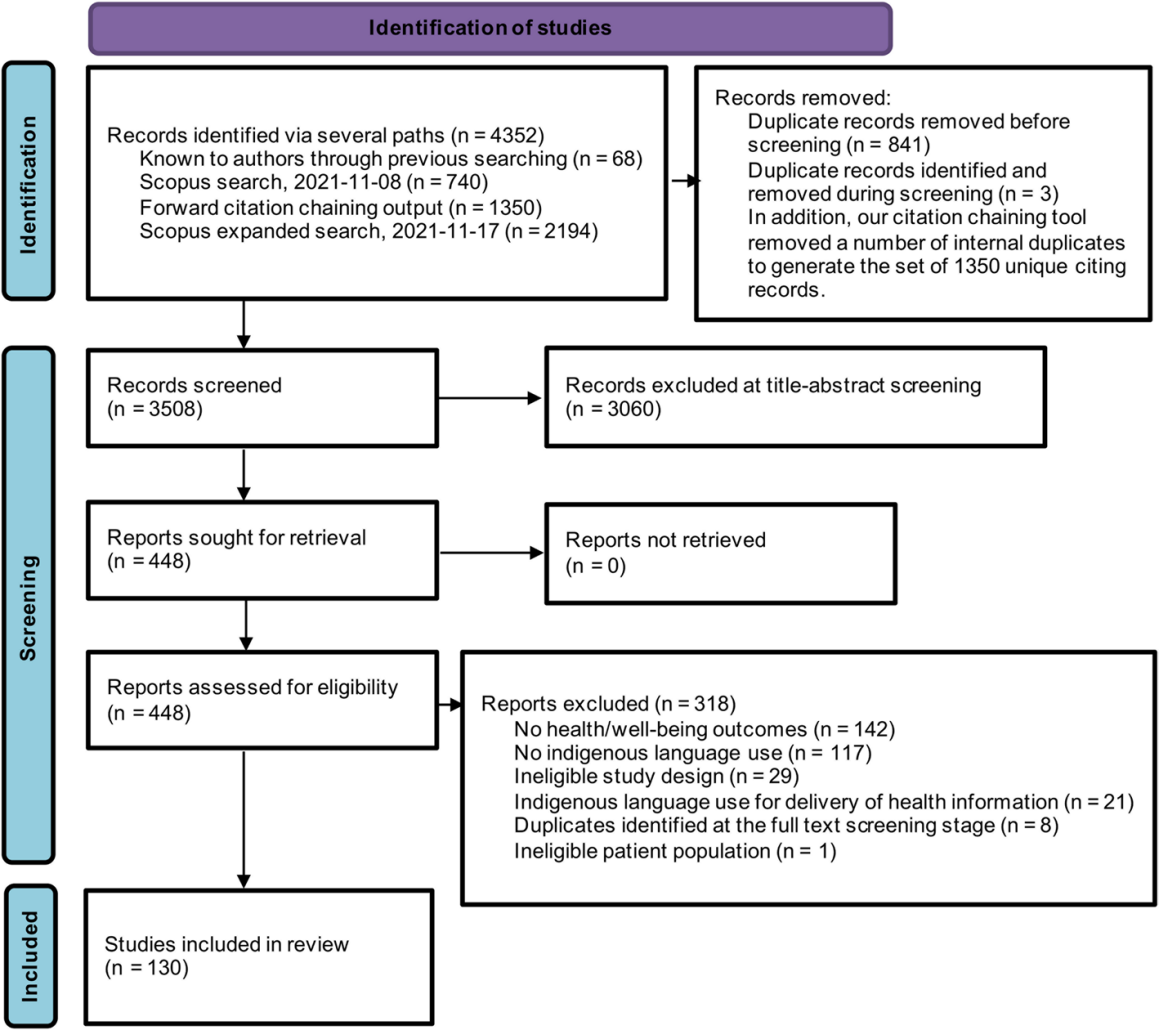
PRISMA 2020 flow diagram of current results. *Adapted from:* Page MJ, McKenzie JE, Bossuyt PM, Boutron I, Hoffmann TC, Mulrow CD, et al. The PRISMA 2020 statement: an updated guideline for reporting systematic reviews. BMJ 2021;372:n71. doi: 10.1136/bmj.n71. For more information, visit: http://www.prisma-statement.org/

The bibliographic database search and citation chaining yielded a total of 360 references for full text review (see Fig. 1). Table 3 shows the characteristics of the 129 of the 130 papers included for the final analysis, (see below for the explanation of the exclusion).

**Table 3:** Effects of Indigenous language maintenance and/or revitalization on health issues. Numbers reflect the study involved. See Table 2 for elaboration of Health Outcomes. Studies were either Qualitative (part a) or Quantitative (part b). Some reports include multiple issues and/or techniques and will thus be listed more than once.

Health Outcome	Positive	Neutral	Negative
a. Qualitative studies			
General	[21], [35], [40], [41], [42], [43], [44], [45], [46], [47], [48], [49], [50], [51], [52], [53], [54], [55]	[56]	
Education	[57], [58], [59], [60], [61], [62], [63]		
b. Quantitative studies			
General	[64], [65], [66], [67], [68], [69], [70], [71], [72], [73], [74], [75], [76], [77], [78], [79], [80], [81]	[21], [82], [83], [84], [85], [86], [87], [88]	[89], [90], [91], [92], [93], [94], [95], [96], [97]
Education	[41], [74], [98], [99], [100], [101], [102], [103], [104], [105], [106], [107]	[73], [108], [109], [110], [111]	[112], [113], [114], [115], [116], [117], [118], [119]
Tobacco	[77], [120], [121]		[122], [123], [124], [125]
Suicide	[126], [127], [128], [129], [130], [131]	[132]	
Alcohol/drugs	[73], [74], [120], [133], [134], [135]	[136], [137]	
Obesity	[77], [138], [139]	[140], [141]	[142], [143]
Diabetes	[144], [145]		
Mental	[107], [146], [147], [148], [149], [150], [151], [152], [153]	[154]	[65], [155]
Crime	[133]	[73], [74]	
Other	[156], [157], [158], [159], [160]	[161], [162]	[159], [163], [164], [165], [166], [167]

It is worth noting that one of the papers cited in the original 2016 review [36] did not pass the abstract screening stage. The degree to which language informed the “enculturation” metric was not obvious from the abstract, so the article was not passed on to full-text review. By comparison, even more straightforward screening processes have been found to have a 3% miss rate for dual screening [37], so this gap is not completely unexpected. It is therefore likely that other relevant articles would have been found had it been feasible to do a full-text review of all articles. A second study that was included in the original study was excluded at the full text stage [38]. The connection between geographic area and language use did not seem as strong in relation to the other studies found in this realist review for inclusion in this paper.

One study was excluded from the tables because there were contradictions between the description in the text and the data in the table [39]. The text claims a negative effect of language while the table shows a positive effect. The table may have had a miscoding that did, indeed, match the verbal description, but that was impossible to assess. Our solution was to exclude the study altogether.

## Results

**Fig. 2:**
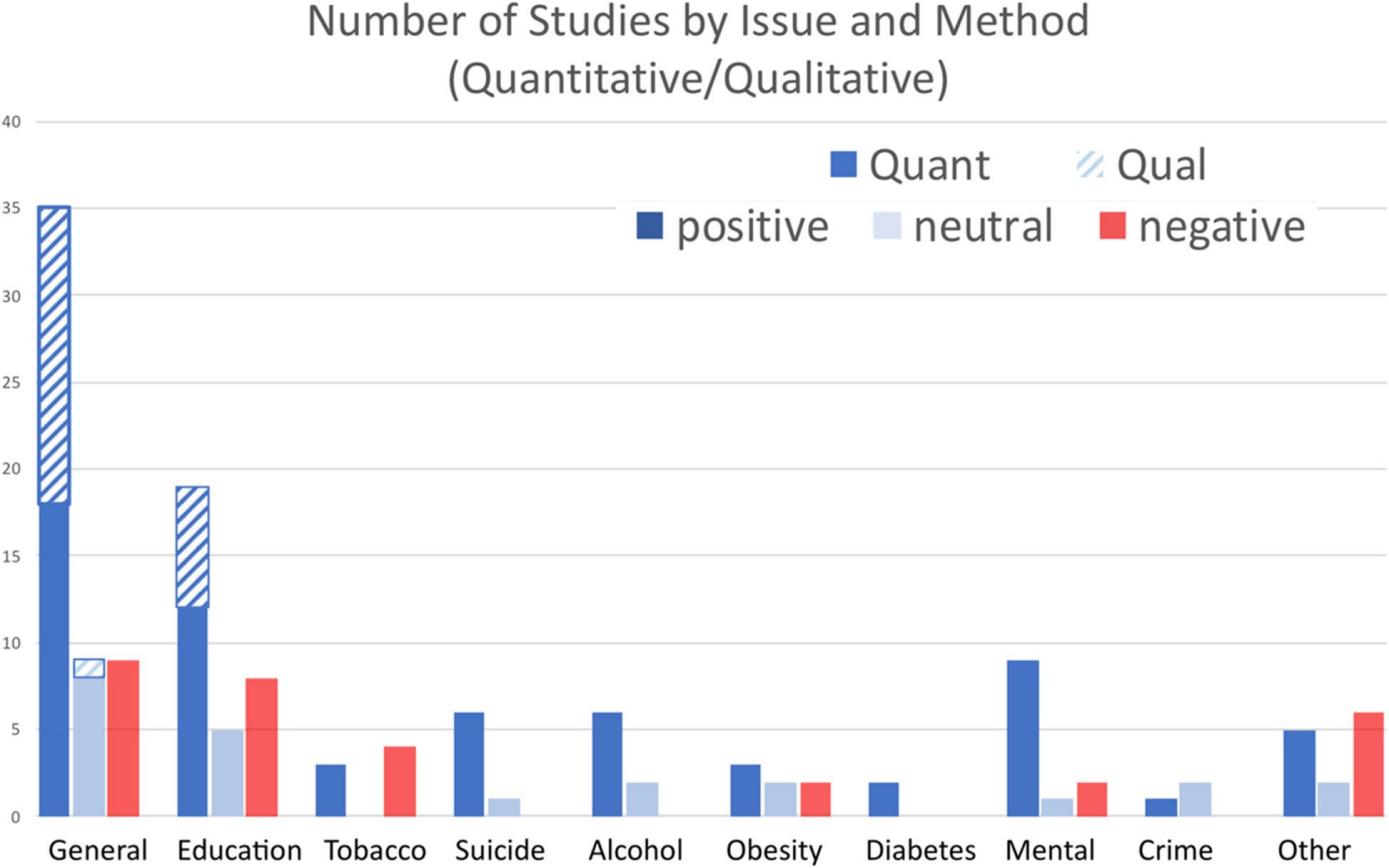
Count of study results in the included studies. Positive outcomes are in dark blue; neutral in light blue; and negative in red

Results indicate that the majority of reviewed articles found a relationship between Indigenous language use and positive health outcomes (*N* = 90, 62.1%); the remainder were fairly evenly divided between neutral (*N* = 24, 16.6%) and negative (*N* = 31, 21.4%). (Quantitative studies reported statistical significance while qualitative ones did not.) The total number of reported effects is larger than the number of citations because some articles reported more than one outcome. Table 3 lists the citation number of the cited studies organized by Health Outcome, Quantitative/Qualitative, and Positive/Neutral/Negative. Several studies addressed more than one issue, or the same issue by both quantitative and qualitative means, and therefore will appear more than once. Fig. 2 presents the counts of the results graphically.

Qualitative results were only found for the categories “General” and “Education.” They were overwhelmingly positive, with no negative results and only one neutral one. Speakers maintaining their language and learners acquiring an ancestral language both report general improvement in health or ability to achieve academic goals. One author even showed improvement before beginning a language program, as she “made a commitment for four years to not drink because [she] wanted to be a good language learner” ([35]: p. 866).

The quantitative studies for those same two categories are generally positive (50.0%), but with more neutral results (21.7%) and the presence of negative results (28.3%). For the “General” negative results, all nine studies mention the high correlation of Indigenous language use and poverty as a potential underlying factor. Many of the “Education” negative results were based on assessments made in the matrix language, not in the Indigenous one. However, there are positive cases in just those same circumstances (e.g., [61, 99, 102, 110]). Because the correlation with poverty is prevalent in the positive cases as well as the negative ones, the positive outcomes are even more impressive.

Reports of the effect of language use on rates of smoking were fairly evenly divided, with three positive outcomes and four negative ones. The former were all based on surveys conducted in the United States, while the latter were performed in Canada. It is possible that the construction of the surveys differed in ways that would skew the results one way or another, or it could be that the geographic difference is a real one. Cultural factors may differ enough between the two countries that the difference is genuine, even though many tribes and bands cross the national border.

The results are even more mixed for obesity, both across studies and within. Here, the geographic difference found for smoking did not appear, as studies of Canadian populations occurred in all three categories, with the US and New Zealand showing positive or neutral results. Within studies, the results can be more mixed than our schematic results indicate. For example, Young [143] found negative associations for women but neutral ones for men. This was attributed to different rates of acculturation, as the socio-economic status (SES) differed between the groups. The recent emphasis on traditional foods is not reflected in these studies, and future developments could be expected to show more positive results due to the frequent incorporation of Indigenous languages into the traditional food movement [168].

The two studies of language use and diabetes, one from the US and one from New Zealand, reported positive correlations. Oster et al. [144] and Teng et al. [145] both assessed Indigenous language use and health status using public records. It is important to note that one study that surveyed diabetic patients from small communities in Mexico [160] was not included in the diabetes category. Instead, it was categorized as “self care” given that is the health outcome that was measured. Therefore, this study appears in the “Other” category. Although we classified its results as negative, the results for language as a main effect were not significant; it was only in combination with poverty that language appeared as a risk factor (Fig. 1, p. 885). As has been the case with other negative results, the correlation of Indigenous language use is at times also correlated with poverty, which itself is independently linked to negative health outcomes. Without that link, the use of language appears to be a protective factor for “self care” among persons with diabetes.

Crime, which includes both arrests and being a victim, was found to have language as a protective factor in one study and a neutral factor in two others. All three cases are from Australia. The positive result [133] was for the experience of violence in remote areas, where speaking the local language may have led to more resilient community connections. One of the neutral results ([74]: 326) was based on conflicting results for strong vs. moderate/weak cultural attachment (to which language use was a major contributor): Strong connections were protective against arrest by the police, but moderate and weak connections led to (nonsignificant) increases in arrests. The other neutral result ([73]: 23), which coded language use more directly, found no effect for ever having been arrested by the police with strong and weak language use, but positive (protective) effects for the moderate language use group. These rather conflicting results suggest the need for more detailed study of the “crime” category, both within Australia and in other regions.

There were 13 studies that addressed other issues. Results were more mixed in these cases. Some of the outcomes did not seem to be strong health indicators (e.g., poverty, less intercourse, cancer screening). Many of these studies were difficult to interpret, relying on high level descriptions of language use (e.g., from census data) or finding marginal results in complex analyses.

The reports for mental health and suicide reduction were largely positive (78.9%), with neutral and negative cases each accounting for 10.5%. Results on suicide have been among the most commonly cited on the issue of language and health. Mental health illness and distress within Indigenous communities is elevated in part given that racism based on tribal identity is often a source of discrimination and degradation in non-Indigenous society. This can relate to risk to physical and emotional health [169]. Cultural connection offers a buffer to the stresses of bias and discrimination by offering connection, support, and culturally specific ways to address negative experiences. Overall, however, language use has clear positive benefits on improving mental health for all ages, and in reducing suicide, particularly with youth.

## Discussion

The published literature substantially supports the hypothesis that active use or learning of an Indigenous language has positive health benefits. The majority of studies (62.1%) indicate positive effects, while a minority show negative effects (21.4%). This is critical information for language programs and health programs alike given that many Indigenous communties face persistent public health crises, as well as impending language loss. These results follow major trends demonstrating the importance of enculturation. For instance, cultural tailoring of health programs or the use of “culture as treatment” itself produce positive health outcomes within Indigenous communities [168, 170–172]. The issues range from cardiometabolic disease to mental health and to substance abuse. In other words, traditional cultural beliefs and practices are health promoting and their absence poses serious health risks to Indigenous communities.

Although qualitative studies, especially those based on self-report, may have an intrinsic positive response bias, we encountered no negative qualitative reports in our review. Those who feel that learning the language did not help them, or even set them back, may be less likely to be located and report in this kind of literature. However, qualitative reports offer a richness to data that is less often found in quantitative data and these may be important articles to highlight when moving forward in learning about mechanisms of improved health via language maintenance and revitalization.

Most of the negative effects on health arise from strong correlations between Indigenous language use and confounding factors such as SES. Considering that poverty has a well-established negative influence on health (e.g. [173, 174]), such an outcome is not surprising. What is surprising is that the majority of reviewed studies show a positive effect in health outcomes despite the correlation of language use with poverty. Thus, Indigenous language use could be a protective factor for health and well-being for those experiencing poverty.

One specific area that is well-represented in both positive and negative findings is tobacco use. There were 3 studies that found positive influences and 4 that found negative influences of language use. The negative studies were all based on large-scale survey data, and the others were based on small-scale surveys. Three of the four negative studies examined the 2012 Aboriginal People’s Survey [175]. This survey did not list tobacco use as a potential traditional activity (p. 55), even though some cigarette smoking may be considered a cultural practice [176]. The Survey’s assessments of tobacco use included three responses, “smoking frequency, age began smoking, exposure to second-hand smoke in the home” (p. 57). These limitations may have biased the results toward negative interpretations. Small-scale surveys, on the other hand, might elicit a desire on the part of the respondent to appear more healthful than is accurate. Further, even though the reports were specifically about cigarettes, it is not clear whether Ryan et al. ([123]: 115) found no correlation between degree of cultural practice and smoking, while Wolsko et al. [121] found that traditional culture was correlated with less cigarette consumption (though greater smokeless usage, as found in other studies of Yupik populations). One positive outcome for tobacco use [121] is based on the use of “iq’mik,” a smokeless, chewing mix of tobacco, moss, and other ingredients. This has been found to lower, rather than raise, biomarkers for ill effects of tobacco [177]. While it is possible that this particular tobacco use has positive health effects, for the purpose of this article, we will continue to code other tobacco use as negative in line with the recommendations of major health organizations (e.g., American Heart Association). Hopefully, more research around Indigenous specific practices, uses, and types of tobacco will be able to more clearly demarcate harmful versus protective uses of tobacco [176].

Some of the negative and null results for education relied on testing L1 speakers in the matrix language, in these cases, English or Spanish. Although this can appear as an unfair assessment of a student’s progress, there are also positive cases, especially for L2 speakers of Hawaiian [178] and Myaamia [41]. English language results were better for L2 students compared to those who were using only English in all cases. This may be due to the positive cases’ examining students learning the Indigenous language as a second language (L2) and are already competent in the primary language (L1), while the negative studies were largely based on first-language speakers (L1) who are learning English or Spanish as an L2 for the first time. The educational environment can also be expected to differ in these two cases, with more effort (and therefore support) being required for the overall school environment in the L2 case, in which the new (Indigenous) language must be deliberately implemented. Those L1 speakers in an L2 monolingual school environment, on the other hand, can be seen to impose an extra burden on the teachers because they are likely to have weaker skills in their second language than students in the same class who have that language as their first one. Given this interpretation, there is further support for L2 learning as it has been shown to improve, or at least not impair, L1 (matrix language) achievement.

Language revitalization can be performed at widely varying levels of funding. For example, a Canadian study found that an average of $5-6 million (Canadian) per year ($4-5 million US) would support language maintenance and revitalization for one community [179]. Programs aimed at individual health issues can be effective in a more focused way, but they are unlikely to address other health issues. Budgets for health vary greatly by tribe. For example, one of the largest tribes in the country (Cherokee Nation) had a budget of $924.5 million for health and $18 million for language in 2021 [180], while a tribal community in New Mexico spends $1 million per year [181]. Some language revitalization programs began with virtually no money [182] and yet went on to succeed in their language efforts. Others have had initial funding which was not sustained, resulting in the closing of the program [183]. Overall, the cost of revitalization is quite comparable to those health programs addressing a single issue, yet it demonstrates positive effects for multiple health issues.

The largest proportion of positive studies in one area occurred for mental health and suicide prevention. Suicide among American Indians is double the rate of non-American Indians in the US and is a clear public health crisis [184]. The most promising programs directly addressing single issues are the mental health/suicide interventions today that center on Indigenous culture [185–187]. The feeling of connectedness to community and pride in cultural heritage are enhanced by learning or maintaining an Indigenous language. As acknowledged elsewhere, other cultural activities besides language can also improve connectedness and pride. Language is, however, the most definitive and most universal expression of a culture. Cultural activities such as beading, drumming, canoe building, etc., will not be shared by all members of a community. Language can be part of all of them. There are yet to be any studies that directly compare cultural revival with and without language. Based on the literature and the results of this study, our expectation is that adding language revitalization to cultural revival will have a significant and large separate influence on improving mental health.

## Conclusions

As we enter the United Nations International Decade of Indigenous Languages (https://en.unesco.org/idil2022-2032), it is important to assess the specific benefits of Indigenous languages. The results of this survey clearly indicate that Indigenous language use—regardless of proficiency level—has positive effects on health. While further research is needed to understand the mechanisms and most effective practices, Indigenous communities can be confident that their language revitalization programs are worth the effort and cost. Relative to the cost of individual programs directed at each of the health issues studied here, language programs hold the promise of widespread effects from a single program. Indigenous groups have endured decades of relatively poor health outcomes. Language revitalization is both empowering and promising for making significant improvements to the health and well-being of Indigenous communities.

## Supplementary Information

**Additional file 1.**

## Data Availability

Not applicable.

## Footnotes

1 We use the term Indigenous to refer to the Native people of the Americas, Australia and New Zealand (the geographic areas surveyed here). We understand the use of labels has ethical and political implications, and we do not wish to perpetuate the insensitivity of many of these labels. Further, the word Indigenous will be capitalized to indicate that it is being used in this specified sense.
